# Supplementary material for: Direct Synthesis of MOF-74 Materials on Carbon Fiber Electrodes for Structural Supercapacitors
Source: Nanomaterials (Basel). 2024 Jan 20;14(2):227. doi: 10.3390/nano14020227 (PMC10819144; doi:10.3390/nano14020227)
Supplement: Supplementary file 1 [file nanomaterials-14-00227-s001.zip › nanomaterials-2818111-supplementary.pdf]

Supplementary Material

Direct synthesis of MOF-74 materials on carbon fiber electrodes for structural supercapacitors

David Martinez-Diaz [1*], Pedro Leo [2], David Martín Crespo [1], María Sánchez [1], Alejandro Ureña [1]

[1] Materials Science and Engineering Area, Escuela Superior de Ciencias Experimentales y Tecnología, Rey Juan Carlos University, C/Tulipán s/n, 28933, Móstoles, Madrid, Spain
[2] Departament of Chemical and Enviromental Technology, Rey Juan Carlos University, Móstoles, Spain
* Correspondence: david.martinez.diaz@urjc.es

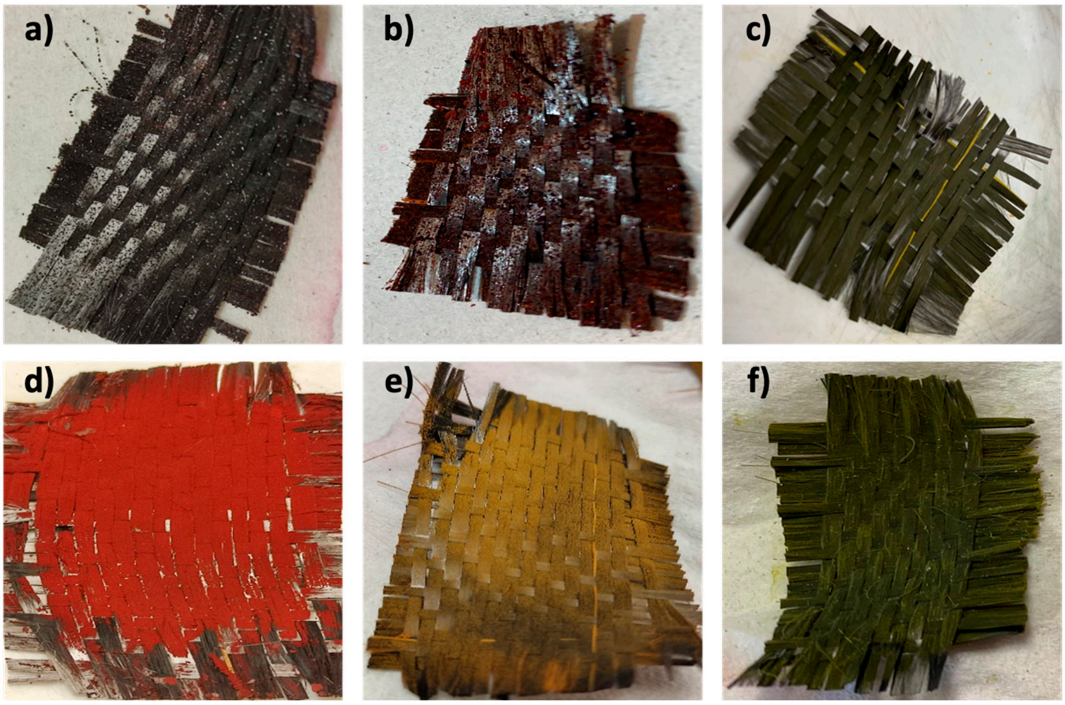

Figure S1. Surface modification of the carbon fiber fabrics with M-MOF-74 Synthesis. M= Mn (a), Co (b), Ni (c), Cu (d), Zn (e) and Mg (f).

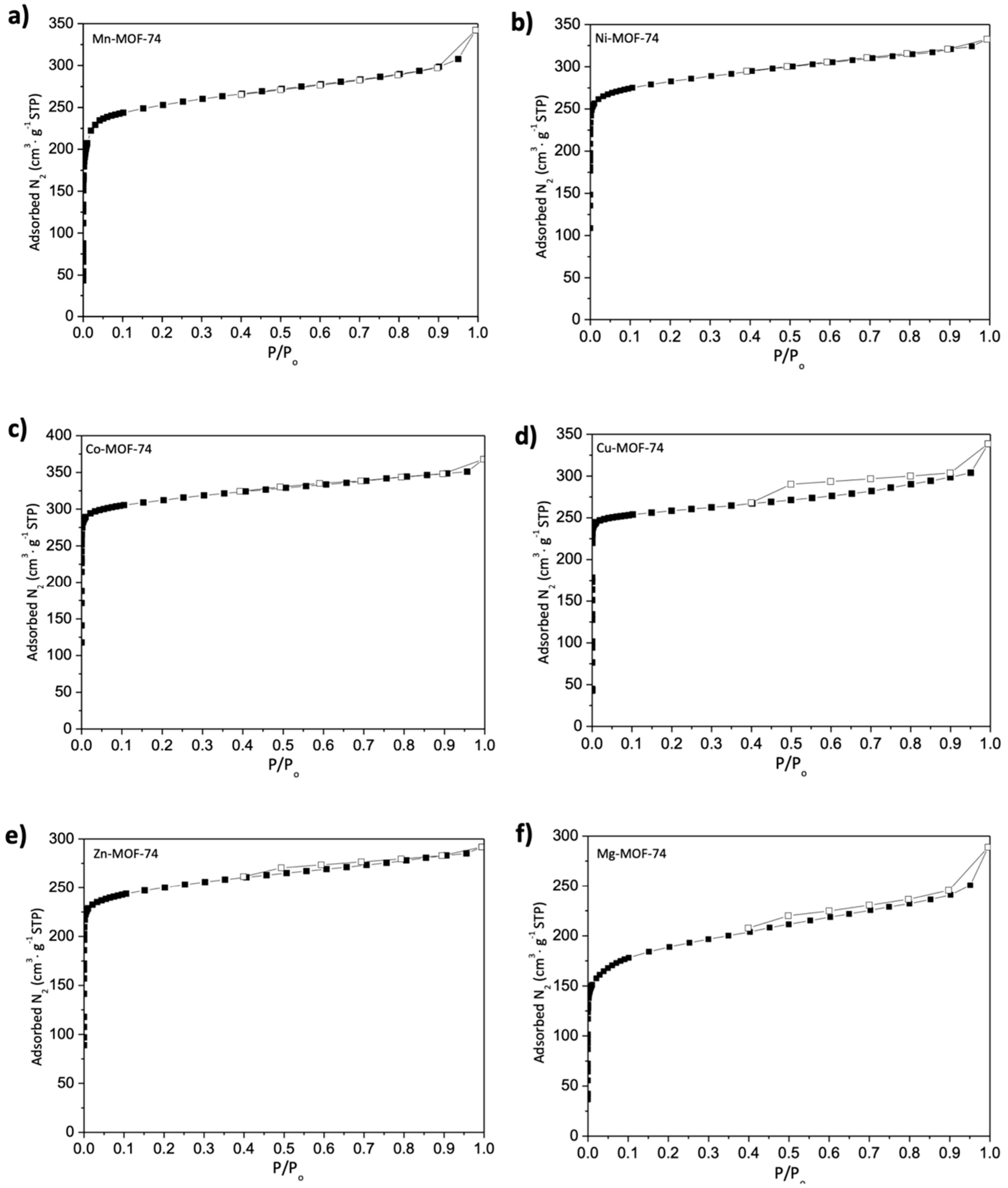

Figure S2. N_2 adsorption/desorption isotherms of MOF-74 family.

Table S1. Specific capacitances for the different used active metals on M-MOF-74 structure used as electrodes (M= Mn, Ni, Co, Cu, Zn and Mg).

Scan rate / Active metal	Specific capacitance (F/g)					
	5 mV/s	10 mV/s	25 mV/s	50 mV/s	75 mV/s	100 mV/s
Mn	0.8847	0.3669	0.1162	0.0394	0.0152	0.0077
Ni	1.1878	0.4777	0.1360	0.0351	0.0085	0.0029
Co	1.9469	0.8463	0.2722	0.0881	0.0307	0.0129
Cu	0.4484	0.1663	0.0510	0.0148	0.0062	0.0024
Zn	0.7568	0.2856	0.0791	0.0221	0.0074	0.0061
Mg	0.5924	0.2293	0.0654	0.0188	0.0058	0.0021
CF	0.0595	0.0223	0.0060	0.0016	0.0005	0.0002

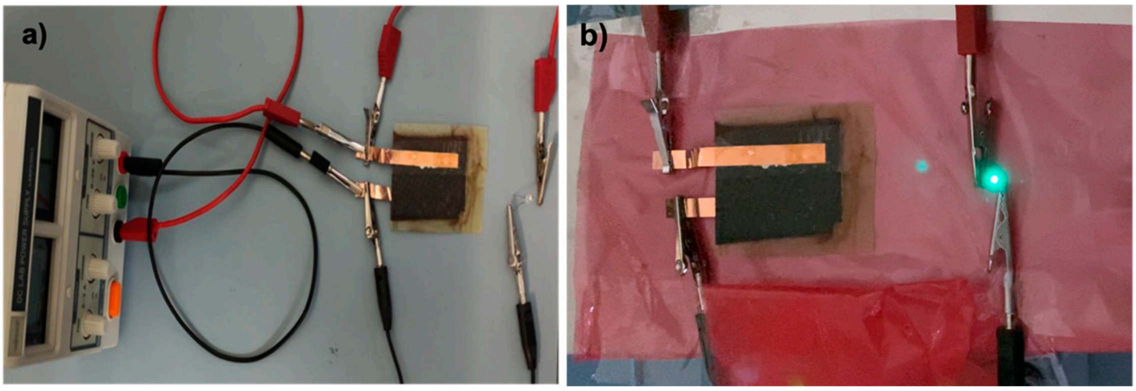

Figure S3. Co-MOF-74 structural supercapacitor charging step (a) and working mode with the LED lighting (b).
